# Associations between happiness with social factors and opioid agonist therapy among people who inject drugs

**DOI:** 10.1038/s41598-025-95967-y

**Published:** 2025-04-06

**Authors:** Clara Lucas Guerra, Jørn Henrik Vold, Christer Frode Aas, Fatemeh Chalabianloo, Else-Marie Løberg, Kjell Arne Johansson, Lars Thore Fadnes

**Affiliations:** 1 Facultad de Enfermería, Fisioterapia y Podología, Universidad Complutense de Madrid, Madrid, Spain; 2 Department of Addiction Medicine, Bergen Addiction Research, Haukeland University Hospital, Bergen, Norway; 3 Department of Global Public Health and Primary Care, University of Bergen, Bergen, Norway; 4 Facultad HM de Ciencias de la Salud, Universidad Camilo José Cela, Villafranca del Castillo, Madrid, Spain; 5 Division of Psychiatry, Haukeland University Hospital, Bergen, Norway; 6 Department of Clinical Psychology, University of Bergen, Bergen, Norway

**Keywords:** Drug discovery, Psychology

## Abstract

The level of happiness is low among patients with chronic mental or physical disorders. However, happiness and its association to sociodemographic and clinical factors remain unknown among patients with opioid use disorder (OUD) and people who inject drugs (PWID). This prospective cohort study aimed to examine self-reported happiness levels in patients with OUD/PWID, changes over time, and its associations with sociodemographic factors, opioid agonist therapy (OAT), and substance use. From 2017 to 2023, 967 patients with OUD/PWID were examined at baseline and one-year follow-up assessments in eight OAT outpatient and municipality clinics in Norway. Happiness was assessed with an eleven-point Likert scale and presented as percentage (from 0% “completely unhappy” to 100% “completely happy”). A linear mixed model analysed associations between exposures and outcomes at baseline and over time. Participants were predominantly men (71%), with a mean age of 43 (SD: 11); 87% received OAT. Mean happiness was 45% of min-to-max (95% CI: 35;54) with an 11% improvement over time. High substance use (-24%, 95% CI: -32; -16) was associated with lower self-reported happiness at baseline, but self-reported happiness increased over time among those with more substance use. Living with someone was linked to higher happiness compared to living alone.

**Supplementary Information:**

The online version contains supplementary material available at 10.1038/s41598-025-95967-y.

## Introduction

Happiness is defined as the subjective enjoyment of one’s life as a whole^1^. Recent survey studies show that happiness has a high ranking in the value hierarchy of the general public^2^. The mean happiness in 2022 based on 134 countries when converted to scale from 0% indicating “completely unhappy” to 100% indicating “completely happy” was 55%^3^. Social support, stable housing conditions, good health, and good economy have been identified as predominant determinants of happiness in general populations^4^. A decrease in happiness could be caused by the determinants of physical and mental health such as diseases, depression, poor perception of health and substance use^4,5^. The mean level of happiness is lower in chronic diseases such as psoriasis (53%)^6^, or multiple sclerosis (46%)^7^. Having mental disorders is related to lower levels of happiness^8^. Patients with mood disorders such as depression report 43% of happiness. Patients with obsessive compulsive disorder report 39%, while patients with substance use disorders (SUD) have been reported to have even lower mean levels of happiness, with 38%^8^, however, there might be wide variations within patient groups.

More than 44% of the patients with SUD suffer from negative mental health^9,10^ and have low health-related quality of life (HRQoL) compared with the general population^11^. Among patients with opioid use disorder (OUD) receiving opioid agonist therapy (OAT), symptoms of mental health disorders are observed among 50% of the patients^10,12,13^, with even higher prevalence if using benzodiazepines, cannabis, opioids and among people who inject drugs (PWID), who are at higher risk for more frequent and severe symptoms^14^. OAT is an evidence-based medical intervention that reduces illicit opioid use and improves patients’ mental and physical health, as well as quality of life^15–18^. A prospective cohort study showed significant improvement in overall HRQoL at one-year follow-up for half of the patients in OAT^11^. OAT has been found to have positive effects on survival and HRQoL, but less is known about the subjective experience of happiness in patients with OUD/PWID. Happiness may be an important marker of the combined outcomes that really matter for the individual patients^1^. To explore what is important for patients with OUD/PWID it is imperative to examine change in happiness over time and how sociodemographic characteristics and clinical variables may impact happiness.

Selecting the right metrics in research is of paramount importance. Validated measures and metrics inform policy decisions and are used to understand the effects of interventions and disease burden, such as survival and mortality^19^, prevalence and incidence^20,21^, comorbidity^22^ or hospitalization rates^23^. Quality of life and quality-adjusted life years can be also assessed with survival and are measures of well-being^24,25^. However, they are not necessarily capturing how people perceive their lives, and happiness measures are needed to contribute to a holistic approach. Recent studies highlight the importance of learning what matters to patients with OUD and emphasize the need to focus on PWID due to their elevated risk of more severe mental health symptoms^26,27^. Participants redefined recovery in terms of improved well-being as opposed to the official definition of total abstinence^28^. Thus, taking a patient-centered approach may contribute to treatment adherence^29^. Besides treatment goals, motivations and expectations of users may play an important role in treatment programs.

The hypothesis of this study is that higher use of substances is linked with lower levels of happiness while OAT could improve happiness levels in patients with OUD/PWID over time. Happiness might also be associated with sociodemographic factors. This prospective cohort study aims to investigate happiness and its change over time using a happiness scale in patients with OUD/PWID and assess how this is associated with sociodemographic factors including sex, age, educational level, housing situation, substance use patterns and injecting substance use and OAT. We also present sub-group analyses for patients receiving methadone or buprenorphine as OAT.

## Methods

### Data source

We used data from a cohort nested to the INTRO-HCV study on patients with OAT/PWID in Bergen and Stavanger, Norway^30,31^. We collected data from April 2017 to January 2023, and recruited patients on OAT from outpatient clinics, as well as patients with various OUDs/PWID receiving primary healthcare from the municipality clinics.

### Data collections

Patients were assessed with an annual health assessment, including happiness scale measurements^1,2^, sociodemographic data, psychological distress, and current substance use. We collected all data using electronic data collection software (Checkware) under the supervision of research nurses. The clinical data were including information regarding the treatment, OAT medication, substance use, and possible comorbid clinical conditions.

### Study sample

The study sample included individuals diagnosed with opioid dependence according to International Classification of Diseases version 10 (ICD-10)^32^, aged 18 years or older, and have given written informed consent to participate in the study. Individuals were eligible for inclusion regardless of the type of OAT medication or administration form. We also included patients who injected drugs, encompassing those using opioids exclusively, opioids in combination with other injectable drugs such as amphetamines, or other injectable drugs, and receiving follow-up from community care clinics in Bergen municipality located near their place of residence. These clinics provided care and some food provision, but no medication.

Patients were excluded if they did not complete the interview or due to missing data.

We included 2202 measurements from 967 patients in the study period. Of these, 94% (908) had opioid dependence and 86% (844) were enrolled in OAT while 12% (123) did not receive any OAT medication. Among 123 patients who not received OAT, 5% (57) had not used opioids in the last 30 days, but most of these had injected stimulants such as amphetamines. In total, 572 had two or more happiness measurements (median of 2, 25–75% of 1–3). The median time interval between health assessment was 14 months (interquartile range [IQR]: 11–19).

### Measuring happiness

We measured self-reported happiness during the study period using the happiness scale (HS). The HS is a validated self-reported scale composed of a question on “*How happy are you with your life these days?*”^1,2^. The response options were denoted with numbers from 0 (“completely unhappy”) to 10 (“completely happy”). We dealt with each HS measurement as a score multiplied by 10 ranged from 0–100%. We present HS measurements converted to a percentage scale from min-to-max. A HS measurement was completed in two time points during an annual health assessment. The data collection software only allowed valid responses to the question and prompted empty questions before submission to minimise missing data.

### Definition of study variables, including sociodemographic and clinical factors

The definition of the study variables is presented in Additional file 1.

### Psychological distress

The measure of the psychological distress^33–35^ is presented in Additional file 2.

### Statistical analysis

We used Stat/SE 16.0 (StatacorpTX, USA) for descriptive analysis and linear mixed model analyses, and SPSS version 29.0 (International Business Machines, Chicago, USA) for expectation-maximisation imputation. The threshold for statistical significance was set to *p* < 0.05. In all analyses, we defined time as years from baseline. We dealt with any missing values concerning sociodemographic and clinical factors, including educational level, housing situation, years in OAT, living condition, having children, suicide attempt, and substance use– as ‘missing at random’ when running expectation-maximisation imputation. We identified missing values in 5% in these factors and all were replaced with estimated values by imputation. Linear mixed model analyses were used to investigate whether the sociodemographic and clinical factors including sex, age, level of education, housing conditions, number of years in OAT, OAT medication, living together, having children impacted HS at baseline and over time. We specified the linear mixed model as a random intercept fixed slope regression model. The estimator was set to restricted maximum likelihood. The predictor variables were kept constant to the value held at baseline. The full information maximum likelihood ensured that all available HS sum score measurements were used. Additionally, we presented sub-group linear mixed model analyses for patients with OUD/PWID using methadone or buprenorphine, respectively, adjusted for sociodemographic and clinical factors. To assess the strength of associations between HS, SCL-10 and substance use, correlation analysis was carried out. The tool Sankeymatic (sankeymatic.com) was used to generate a Sankey diagram for graphical presentation of the change in HS over time.

### Ethics approval and consent to participate

The study was reviewed and approved by the Regional Ethical Committee for Health Research West, Norway (REK Vest 2017/51). Each patient provided written informed consent prior to enrolling in the study. All research was performed in accordance with relevant guidelines/regulations.

## Results

A total of 71% of the patients were men and the mean age at baseline was 43 years (SD 11 years) (Table 1). The mean duration of OAT among patients in the study was 8 years (SD 6 years). A total of 87% received OAT, 54% receiving buprenorphine-based medications and 32% receiving methadone. Related to education, 49% had less than 10 years of completed education and 96% of the patients did not have formal paid work. Further, 13% lived under unstable housing conditions and 65% lived alone, and 53% had children of which 46% were under 18 years old. During the last month leading up to the first health assessment, the most common non-prescribed substances consumed were cannabis (64%) and alcohol (55%), followed by non-prescribed benzodiazepines (54%), stimulants (42%) and illicit opioids (28%). A total of 42% of the patients reported to have injected a substance at least once during the last month.

**Table 1:** Sociodemographic and clinical characteristics at baseline.

	Patients (*n* = 967)
**Sex**,** nº (%)**	
Male	685 (71)
Female	282 (29)
**Age groups**,** nº (%)**	
< 25	35 (4)
25 - <40	353 (36)
40 - <60	518 (54)
≥ 60	61 (6)
**Duration of OAT treatment in years**,** mean (SD)**	8 (6)
**Sociodemographic variables**,** nº (%)**	
10 years of education or less	475 (49)
Not formal paid work	929 (96)
Stable housing^a^	845 (87)
Living alone	632 (65)
Having children	507 (53)
Having children < 18 years	209 (46)
Injected drugs last 30 days	306 (42)
**Current OAT medication**,** nº (%)**	
Methadone	310 (32)
Buprenorphine	515 (53)
Other	142 (15)
**Non-prescribed substance use during the past 30 days**,** nº (%)** ^b^	
Alcohol	498 (55)
Benzodiazepines	484 (53)
Cannabis	579 (64)
Opioids	257 (28)
Stimulants^c^	377 2)

^a^Stable housing included living in owned or rented housing or at an institution during the last 30 days.

^b^Each substance class was dichotomized as 0 (no used in the past 30 days) or 1 (used in the past 30 days) and included only non-prescribed substances.

^c^Amphetamine, methamphetamine or cocaine.

**Fig. 1:**
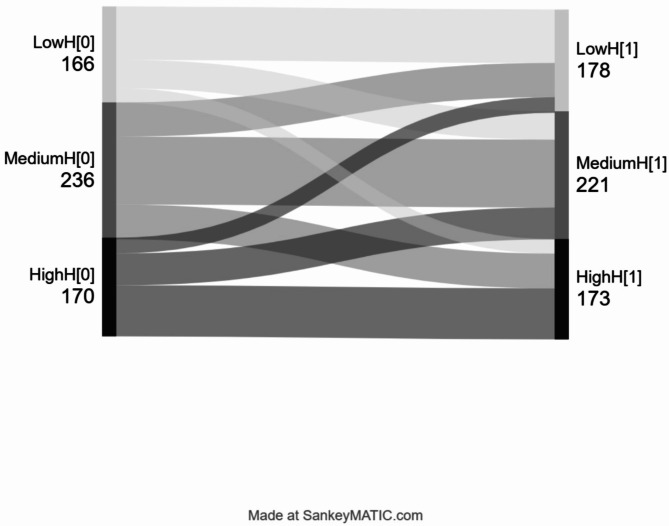
Sankey plot presenting proportion changing between low (light grey), medium (medium grey), and high (dark grey) levels of happiness at baseline and the subsequent yearly follow-up (*n* = 572).

Self-reported happiness was 45% (95% confidence interval [CI]: 35;54) at baseline. Among the 572 participants, 166 (29%) had low self-reported happiness (< 30%), 236 (41%) had medium self-reported happiness, and 170 (30%) had high self-reported happiness (> 70%) at baseline. Among the participants who had low or medium self-reported happiness at baseline, 317 (79%) remained with low or medium self-reported happiness, while 85 (21%) reported an improvement to high degree of self-reported happiness at the next annual follow-up. Inversely, 82 of 170 (48%) participants with high self-reported happiness at baseline had a lower self-reported happiness in the next annual follow-up while 88 (52%) maintained a high self-reported happiness (Fig. 1).

There was an increasing trend in the self-reported happiness over time (11%, 95% CI: 2;19) (Table 2). At baseline, patients living with someone had a higher self-reported happiness (9%, 95% CI: 6;12) than those living alone. More use of substances (-24%, 95% CI: -32; -16) was associated with lower self-reported happiness at baseline, but the time trend was inverse with improved self-reported happiness over the time. Receiving methadone (-12%, 95% CI: -20; -6) or buprenorphine (-12%, 95% CI: -19; -5) as OAT medications were associated with decreasing self-reported happiness over time compared with not receiving OAT medication, but these were balanced by the inverse general time trends.

**Table 2:** Adjusted linear mixed model of the association between self-reported happiness (ranged 0–100%) and sociodemographic and clinical factors at baseline and over time (*n* = 572).

	Baseline coefficients (95%CI)	Annual time trend coefficients (95%CI)
Time trend per year		*11 (2; 19)*
**Age 18-<25 years**	**0 (reference)**	**0 (reference)**
Age 25-<40 years	2 (-6; 10)	-1 (-5; 2)
Age 40-<60 years	-2.5 (-11; 6)	-1 (-5; 2)
Age ≥ 60 years	-5 ( -16; 4)	0 (-5; 3)
**Male**	**0 (reference)**	**0 (reference)**
Female	*-3 (-7; 0)*	*1 (0; 2)*
Years in OAT	-0.1 (-0.4; 0.2)	0.1 (0; 0.2)
**No OAT medication**	**0 (reference)**	**0 (reference)**
OAT medication: methadone	-3.9 (-9.8; 1)	*-12 (-20; -5)*
OAT medication: buprenorphine	0.9 (-4; 6)	*-12 (-19; -4)*
**Educational level**: 10 years or less of schooling	**0 (reference)**	**0 (reference)**
Educational level: more than 10 years of schooling	0.7 (-2; 3)	-0.6 (-1; 0.5)
**Stable living condition**	**0 (reference)**	**0 (reference)**
Unstable living condition	-3 (-8.4; 0.9)	0 (-2; 1.9)
**Living alone**	**0 (reference)**	**0 (reference)**
Living together with someone	*9 (5.8; 12)*	0 (-1; 0.4)
**Not having children**	**0 (reference)**	**0 (reference)**
Having children	3 (0; 6)	0 (-1; 1)
Illicit substance use (0: no use, 1: maximum)	*-24 (-32; -16)*	*5.4 (2; 8)*

* Significant results are shown in italics (*p* < 0.05). *CI* Confidence interval, *OAT* opioid agonist therapy.

The correlation between self-reported happiness baseline values and mean SCL-10 measurement was − 0.55, while the correlation with a history of earlier suicide attempt was − 0.14. (Additional file 2, Table S1)

## Discussion

This study is one of the first to examine the level of happiness and its changes over time in a sample of patients with OUD/PWID and how this is associated with substance use patterns, and clinical and sociodemographic factors. We observed lower levels of happiness in patients with OUD/PWID compared with what has been reported in general populations^4^. Significant improvements in happiness was observed at one-year follow-up. However, large variations in self-reported happiness values were observed between individuals, both at baseline and follow-up.

According to the World Happiness Report, Finland occupies the first position with a happiness score of 78% (converted), Norway occupies the seventh position with 73%, while India occupies the 126th position with a happiness score of 40% (95% CI for rank 17–128)^3^. Patients with OUD/PWID in our cohort reported in average considerably lower (45%) than the Norwegian general population, however there was a positive time trend with an increase of 11% during the follow-up period. A possible explanation of these lower values of happiness might be that OUD and injected drug use entail lifelong struggles with SUD, suboptimal health and poverty, which emphasizes the need for comprehensive professional support^36^. Additionally, SUD compromises interpersonal relationships and social functioning, intensifying social exclusion and further diminishing individual’s quality of life and well-being^15,24^.

We observed the level of happiness increased at one-year follow-up. Qualitative studies reveal that OUD treatment might provide opportunities to improve one’s life situation and well-being^37^. It is not only about a recovery from substance use but also from symptoms of mental illness^29,37^. Still, we should interpret these results very cautiously. In this study, the baseline was the time for the first assessments, but not the time for starting in OAT. Thus, it is not a critical point of time. Most of the patients could have been many years in OAT and their levels of happiness might not be a direct consequence of being in OAT. In fact, we observed large inter-individual variabilities in HS values from baseline to the one-year follow-up time point, in both positive and negative directions. A hypothesis for these findings could be that OUD treatment might be understood as a process instead of an event^38^, and that several life events with events of great importance for how people perceive their lives occur frequently.

Our findings are generally consistent with the results of other studies. Bergsma et al. observed a happiness score of 38% in patients with SUD^8^. Other studies reported a happiness mean score of 44% (SD: 7%^39^) in patients with depression and 53% (SD: 5%^40^) in patients with schizophrenia. It can be assumed that a high prevalence of underlying mental disorders and extensive polysubstance use could have contributed to the observed level of happiness in the OUD/PWID population^41,42^. This is in line with our results demonstrating a relatively strong inverse correlation between psychological distress and happiness scores^14^. People diagnosed as having a mental disorder reported less happiness scores than those without a disorder^5,8,43^. Nevertheless, happiness expresses the well-being more than the psychological distress itself^1,4,39^ and provides supplementary information that could be relevant for clinicians and policymakers when providing health care. Higher symptom severity in patients with major depressive disorders is associated with lower happiness scores, and, inversely, higher levels of self-reported happiness are predictive of recovery^5,44^. Higher levels of happiness has been reported to be associated with decreased use of both cannabis and other substances in patients with OUD^45^. Thus, measuring happiness might be a predictor of recovery from OUD and injected substance use.

Living with someone was the sociodemographic factor that substantially was associated with higher happiness scores. Happiness is driven by the fulfilment of basic physical and social needs. Living with a partner and maintaining social connections are essential for fulfilling these social needs and are significant determinants of happiness^46^. Research has shown that cohabitation is a significant positive determinant of happiness in the general population, with studies indicating that living together with a partner is associated with increased subjective happiness, where social support and emotional connection are key factors^4,46^. However, this was not correlated to happiness values over one year in our study.

This might suggest that although living arrangements provide immediate emotional and social support, their long-term impact on happiness in individuals with OUD/PWID may be limited by other persistent challenges, such as compromised interpersonal dynamics, social exclusion, and the ongoing psychological burden of substance use. Substance use disrupts these relationships, undermining social functioning and contributing to reduced happiness^8,9,16^.

Higher levels of substance use were correlated with lower levels of happiness at baseline, although this association was reduced over time. Thus, one might hypothesise that unhappiness to larger degree influence substance use among people with OUD/PWID than substance use influence happiness. However, this must be interpreted with caution as this is inferred from the longitudinal trends and could be influenced by confounders and biases.

We observed a negative correlation between self-reported happiness, psychological distress and suicide attempts. Psychological distress and suicide attempts are associated with the use of substances during OAT^47^. Aas et al. found that 65% of people with SUD have symptoms of mental health disorders and psychological distress, besides, other studies showed that the impact of comorbid mental disorders on suicide attempts among patients with SUD is significant^14^. However, some of these symptoms are associated with drug use and might change during treatment. This is consistent with other studies showing reductions in the range of mental health symptoms after admission to inpatient or in outpatient OUD treatment, or in residential programs^48,49^. Thus, the reduction in substance use during OAT could reduce psychological distress and, consequently, suicide attempts. These improvements could bring about an increase in the perception of happiness throughout OAT.

The sociodemographic and clinical factors such as sex, age, housing conditions, having children, number of years in OAT or level of education were not substantially associated to happiness in the OUD/PWID population. Our findings are consistent with prior research, showing no differences in happiness across sex or age groups^51^. For HRQoL among people with SUD in contrast, some HRQoL differences have been observed with lower levels of HRQoL in females and older people^11^. Quality of life is also a measure of well-being; however, it might be more linked with a combination of both somatic and mental health. Happiness, defined as the “subjective enjoyment of one’s life as a whole”^1^, might be more related to how people perceive their lives and being happier might be a possible predictor for lower substance use^48–50^, which is strongly correlated with lower levels of psychological distress and a decreased risk of suicide attempts^47,49,50^. For patients with OUD/PWID, happiness might be more directly related to wellbeing than to sociodemographic conditions. Measuring happiness might not only be a predictor of wellbeing, but also an indicator of how well the OAT programs are tailored to the user preferences^5,28,51^. Policymakers could take this into consideration when planning health services, but it needs to be interpreted with some caution and further research is necessary to confirm causal direction of changes in happiness scores in patients with OUD/PWID.

This study has strengths and some limitations. The strength of this study is the large sample size of long-term patients with OUD/PWID at baseline as well as a cohort design, a group usually hard to reach. However, there are also some limitations. The baseline was typically conducted after the initiation of OAT, with patients having been in OAT for a mean duration of 8 years (SD 6 years) thus, the time trends should be perceived as trends over time in patients with stable treatment situations.

Comparing happiness with and without OAT must therefore be considered with caution. Most patients were recruited from OAT outpatient clinics, which could affect the generalizability of our results to other OUD/PWID populations. Moreover, the health assessments were conducted at varying time intervals. This may complicate the interpretation of the observed substance use changes from baseline. Besides, the HS has not been specifically validated for patients with OUD/PWID yet. Finally, for the linear mixed model analysis we considered substance use as a fixed effect and did not account for changes in substance use over time.

## Conclusions

This study shows considerably lower happiness among people with OUD/PWID compared to the general Norwegian population. A negative time trend of happiness was found during OAT, however, there were substantial inter- and intra-individual variations over time. Higher levels of happiness were correlated with lower levels of psychological distress and associated lower initial levels of substance use. The level of happiness increased over the treatment period, although did the use of substances also, meaning that there are other factors than substance use that may result in unhappiness. This emphasizes the importance of measuring and taking happiness into account in the follow-up of people with OUD/PWID. Policymakers, researchers and clinicians could take this into consideration when planning health services.

## Electronic supplementary material

Below is the link to the electronic supplementary material.

Supplementary Material 1

Supplementary Material 2

## Data Availability

Data generated or analysed during this study are included in this published article and its supplementary information files. The identity variable is anonymized.
