# Supplementary material for: Associations between happiness with social factors and opioid agonist therapy among people who inject drugs
Source: Sci Rep. 2025 Apr 6;15:11790. doi: 10.1038/s41598-025-95967-y (PMC11973160; doi:10.1038/s41598-025-95967-y)
Supplement: Supplementary file 1 — Supplementary Material 1 [file 41598_2025_95967_MOESM1_ESM.pdf]

Associations between happiness with social factors and opioid agonist therapy among people who inject drugs.

Clara Lucas Guerra (MSN),[1,2,3,4*] Jørn Henrik Vold (MD, PhD),[2,3,5] Christer Frode Aas (MD, PhD),[2,3,5] Fatemeh Chalabianloo (MD, PhD),[2,3] Else-Marie Løberg (PhD),[5,6] Kjell Arne Johansson (MD, PhD), [2,3] Lars Thore Fadnes (MD, PhD).[2,3]

[1] Facultad de Enfermería, Fisioterapia y Podología, Universidad Complutense de Madrid, Madrid, Spain

[2] Bergen Addiction Research, Department of Addiction Medicine, Haukeland University Hospital, Bergen, Norway

[3] Department of Global Public Health and Primary Care, University of Bergen, Bergen, Norway

[4] Departamento de Enfermería, Facultad de Salud, Universidad Camilo José Cela, Villafranca del Castillo, Madrid, Spain.

[5] Division of Psychiatry, Haukeland University Hospital, Bergen, Norway

[6]Department of Clinical Psychology, University of Bergen, Bergen Norway

*Corresponding author: Clara Lucas Guerra (C/Rafael Juan y Seva 6, 1ºB 28028 Madrid, Spain) 0034689593725; claraluc@ucm.es.

Additional file 1. Definition of study variables, including sociodemographic and clinical factors.

We defined baseline for patients as the first annual health assessment that included a happiness scale (HS) measurement when we listed the health assessments chronologically. We dealt with each HS measurement as a score multiplied by 10 ranged from 0% to 100 %. We defined being on opioid agonist therapy (OAT) according to whether patients received buprenorphine or methadone (OAT opioids) at baseline or not. We dichotomized educational level into two classes, those who completed or not 10 years of schooling were categorized as "10 years or less of schooling" and those who had completed more than 10 years of schooling were categorized as "more than 10 years of schooling".

Patients' housing situations in the 30 days prior to the HS measurement were classified into two groups: "stable" and "unstable." The latter category involved patients who had lived on the street, at temporary camping sites or with friends or family during the past 30 days. Others who had a more permanent residence were classified as having a stable housing situation such as patients living in owned or rented housing or at prison.

We categorised living together into two groups: `living alone´ or `living with someone´ according to the living condition at the health assessment. Having children was classified into two groups: `no children´ and `children´. Furthermore, suicide attempt was classified as `no suicide attempts´ and `at least one suicide attempt´ during their lifetime.

Substance use for the last 30 days leading up to the first health assessment was measured for each non-prescribed substance class including alcohol, benzodiazepines, cannabis, opioids (opioids received in OAT were not included) and stimulants (amphetamines or cocaine). Each substance class was answered on a scale ranging from zero to four points, where zero represents "never", one represents "less than one day per month", two represents "one to three days per week", three represents "more than three days per week", and four represents "daily". The substance scores (0-4) were handled separately for each substance class and were additionally summarized as a sum score ranging from

0 to 20 points (for all substance classes) to simplify the scales and make it easier to interpret the results of different substance classes. The substance sum score was divided by 20, ranging from 0 (no substance use) to 1 (daily use of all substances).

Additional file 2.

Psychological distress was measured using the Hopkins symptom checklist (SCL-10), a validated and self-administrated questionnaire.[33] It involves ten items which are each scored on four dimensions from not bothered at all (item score = 1) to extremely bothered (item score = 4). Scores were summed and divided by the number of items answered to obtain the mean item score. A mean score of 1.85 for SCL-10 has been recommended as a threshold for indicating substantial mental health distress.[33-35]

Table S1. Correlation between happiness level, SCL-10 mean and suicide attempt.

	**Happiness**	**SCL 10 mean**	**Suicide attempt**
**Happiness**	1.000		
**SCL 10 mean**	-0.5594	1.000	
**Suicide attempt**	-0.1459	0.2333	1.000

These results are based on the baseline values of happiness, SCL-10 mean score (mean of the items answered), and conducted suicide attempts ever.

The suicide attempt variable is coded categorically 0 (never conducted suicide attempts) and 1 (at least once conducted suicide attempt).
